# An electron microscopic study of the archaeal feast/famine regulatory protein: 6. The cylindrical assembly form

**Published:** 2004-05-01

**Authors:** Sanae A. Ishijima, Lester Clowney, Masashi Suzuki

**Affiliations:** *) National Institute of Advanced Industrial Science and Technology (AIST), AIST Tsukuba Center 6-10, Higashi, 1-1-1, Tsukuba, Ibaraki 305-8566, Japan; **) Japan Science and Technology Agency (JST), Core Research for Evolutionary Science and Technology (CREST), Honmachi, 4-1-18, Kawaguchi Center Building, Kawaguchi, Saitama 332-0012, Japan

**Keywords:** cryo-electron microscopy, Fourier filtration, image processing, selected-area electron diffraction, 3D reconstruction, transcription factor

## Abstract

Using a microcrystal of the feast/famine regulatory protein (FFRP) pot0434017 (FL11), a cryo-electron micrograph was taken, showing a projection of cylinder-like assemblies packed parallel to each other. This electron micrograph was processed in the Fourier space by selecting spots reflecting the packing and, in addition, those reflecting stacking of units inside the cylinders. Twenty seven subimages were selected, each containing three cylinders of 24 discs each, running nearly parallel to each other. By averaging these images and in combination with another average showing a different view [Ishijima, S. A., Clowney, L., and Suzuki, M. (2004) Proc. Jpn. Acad., Ser. B **80**, 183–188], some details of the 3D structure of the cylindrical assembly form are discussed.

## Introduction

Various biophysical and biochemical methods have been applied for studying 3D structures of biomolecules and their assemblies. Among all these methods X-ray crystallography1) is the most reliable, able to determine accurate and detailed (i.e. atomic) 3D structures, if crystals of good quality are obtained. Another method of nuclear magnetic resonance (NMR) has also been established,2) having been applied to proteins in solution. However, the molecular weights of the object proteins are still largely limited, practically, to ~20 k or smaller. Thus, not the whole proteins but domains are usually studied: this method is not suitable for studying large assemblies.

The third and currently the final concrete method is electron microscopy.3)–6) Electron microscopy (EM) is free from size limitations. It can be used for studying proteins in crystals 3),4) as well as proteins in solution3),5),6) when kept in amorphous ice7) at a liquid nitrogen or helium temperature. The problem is the limitation in the resolution: usually the resolution achieved for biomolecules is not much better than 10–20Å, with some notable exceptions.8),9)

In addition, careful considerations are necessary while interpreting densities in EM images. The density obtained by X-ray crystallography corresponds clearly to that of electron distribution in the object. Biomolecules are composed of light atoms such as C, H, O and N, and their EM images are, however, created by weak elastic or inelastic electron scattering from, and weak absorption by, the objects.10) The contrast transfer function, resulting from defocusing used for enhancing the phase contrast, will affect this process, differently depending on the spatial frequencies, thereby further complicating the interpretation.4),10) For this reason only, it is important to evaluate two results obtained by EM and X-ray using the same objects.

**Fig. 1:**
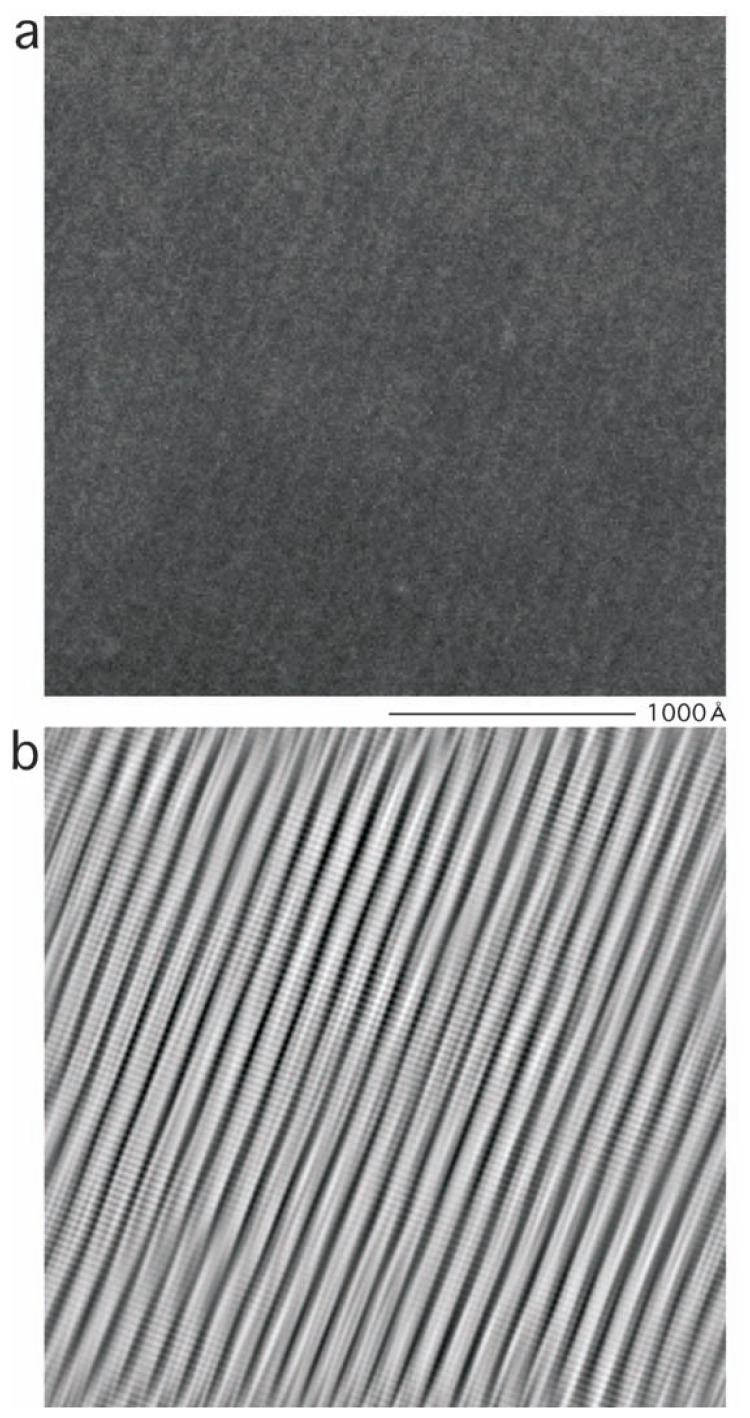
Part (256 × 256 pixels) of electron micrograph 1 recorded at the original magnification of 22.1 k using a CCD camera (a) and an improvement by Fourier filtration (b). The scale 1,000 Å is shown.

While studying the structure and function of a group of bacterial transcription factors, the feast/famine regulatory proteins (FFRPs),11)–22) an FFRP, pot0434017 (FL11) from the hyper-thermophilic archaeon *Pyrococcus* sp. OT3, was crystallized into cylinder-like assemblies.12),22) Using fully grown crystals, the atomic 3D structure was determined by X-ray crystallography. 12) While, smaller crystals, naturally developing 16) or artificially made by sonication,13) were used for cryo-electron microscopic studies. A projection of cylinders to “pencil-like” densities, has been obtained, all such densities related by a hexagonal symmetry (i.e. the top view).13) In this paper a different view (Fig. 1a) of cylinders running side by side (i.e. the side view, although tilted) is analyzed in order to discuss the 3D structure of this assembly form by EM.

## Cryo-electron microscopy

Crystals of protein FL1112) were sonicated,13) to produce microcrystals. A solution, 4 μl, containing these microcrystals13) was placed on a holey copper grid (300 mesh and carbon-coated, Electron Microscopy Sciences Co.). In order to minimize damage caused by dehydration or electron irradiation, the grid was quickly frozen in liquid ethane into an amorphous ice state, using a freezing apparatus (EM CPC, Leica) and maintained at a near liquid nitrogen temperature using a holder (CT3500, Oxford), while an electron microscope (Tecnai F20, FEI) was operated at 200 keV: i.e. the method of cryo-electron microscopy.7)

By irradiating with electrons at a low dosage (~5 electrons/Å^2^), an electron micrograph (here referred to as electron micrograph 1) was recorded at a magnification of 22.1 k, using a charge coupled device (CCD) camera (794IF, Gatan, 1,024 pixels × 1,024 pixels). With this magnification, each CCD pixel covered an area of 10.75 Å × 10.75 Å. In order to minimize chromatic aberration, electrons that had lost no energy (i.e. 200 keV ± 10 eV) were selectively focused,5) using an energy filtration device (GIF200, Gatan). The CCD camera and the energy filter were operated using the Digital Micrograph package (Gatan). Approximately a quarter of the whole area was re-recorded by CCD at a higher magnification, 44.9 k, each pixel covering 5.27 Å × 5.27 Å (refereed to as electron micrograph 2).

**Fig. 2:**
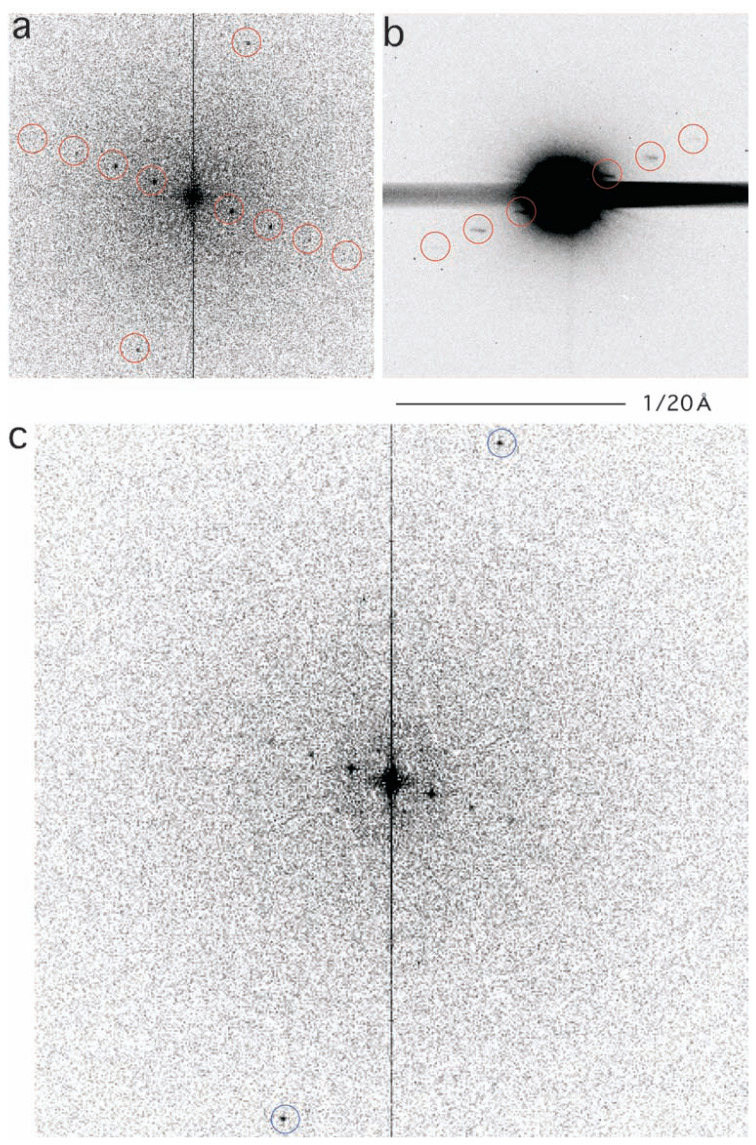
Fourier transforms of electron micrographs 1 (a) and 2 (c), compared with electron scattering from the same area selected (b). Spots used for inverse Fourier transform, thereby producing Fig. 1b, are circled in red in (a). In (b) diffractions reflecting the 113 Å periodicity are circled. In (c) the two spots at the space frequency 1/(13.5 Å) are circled. The scale 1/(20 Å) is shown.

Electron diffraction was also recorded by focusing on the back focal plane of the objective lens (Fig. 2b). The area selected was of the diameter of ~1 μm, and thus was slightly smaller than what was covered by electron micrograph 1.

## A Fourier-filtered image of cylinders

In electron micrograph 1, cylinders, ~100 Å in the diameter, were found running side by side. This packing was clearest near the center, inside an area of 256 pixels by 256 pixels (Fig. 1a), i.e. one sixteenth of the whole electron micrograph.

When a Fourier transform of Fig. 1a was calculated using the digital micrograph package (Fig. 2a), a series of spots were identified in a line with a periodicity of 1/(113 Å), reflecting the separation of the cylinders. Perpendicular to this line, a pair of spots were identified (also circled in Fig. 2a), reflecting a repetition of some form of units along the cylinders with a periodicity of 29 Å. These horizontal and vertical spots were found with various areas in electron micrograph 1, although other types of weaker spots were occasionally found with particular areas only.

Those spots circled in Fig. 2a were thus selected, and the inverse Fourier transform was calculated (Fig. 1b). By this Fourier-filtration,23) some types of noise were removed, showing clearly the parallel packing of cylinders, and, in addition, stacking of “disks” inside the cylinders.

## A combined view of cylinders

**Fig. 3:**
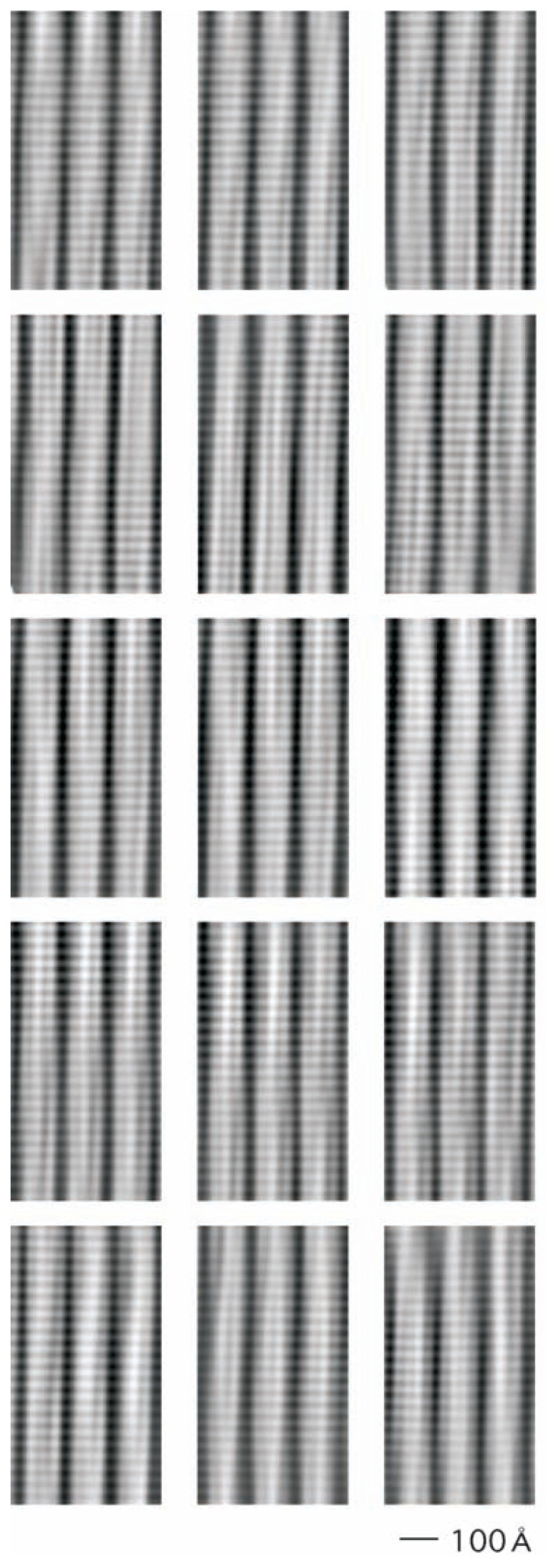
Subimages selected from the Fourier-filtered electron micrograph (Fig. 1b). The scale 100 Å is shown.

**Fig. 4:**
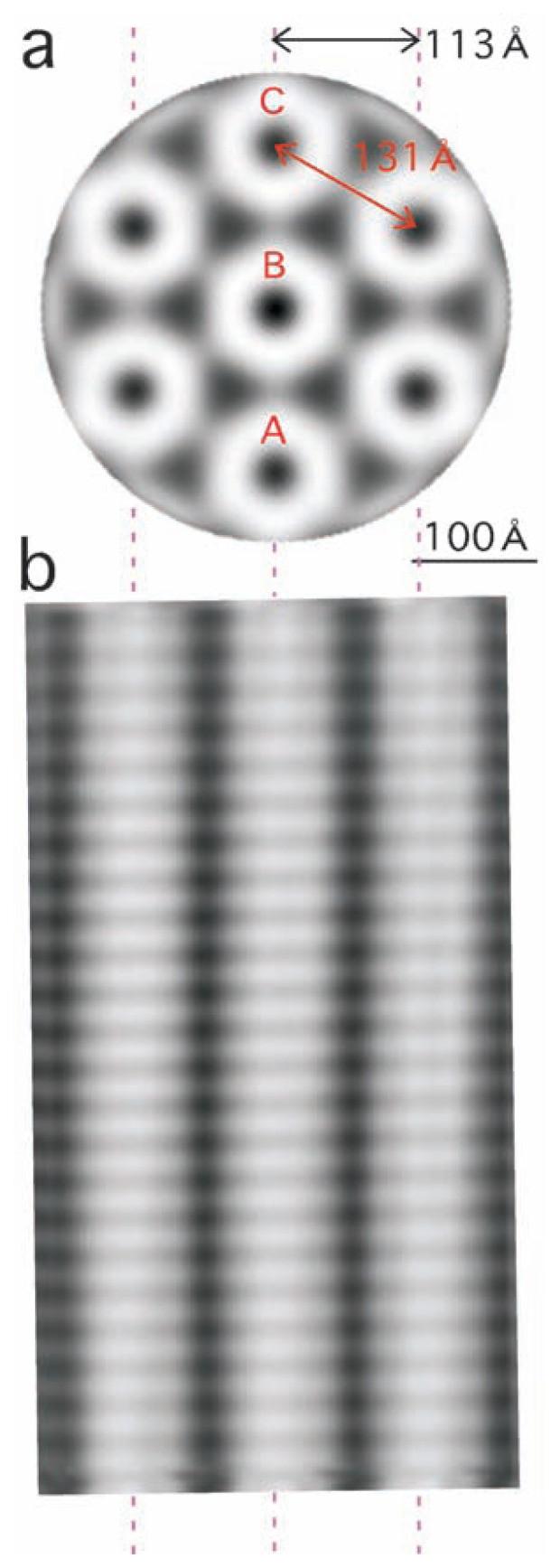
The average of 27 subimages (b) in comparison with another average, showing a different view (a). (a) is a reproduction of Fig. 4d in ref. 13). The scale 100 Å is shown.

From the Fourier-filtered micrograph (Fig. 1b), 27 subimages were selected, in each of which three cylinders, each containing 24 disks, were running nearly parallel to each other: 15 of the 27 are shown in Fig. 3. When these images were averaged with manual adjustments (Fig. 4b), bridges connecting disks between cylinders were more clearly seen.

In Fig. 4b the centers of nearby cylinders are separated by 113 Å. This number coincides with the separation between layers of units, when cylinders are projected as “pencil cross-sections” (i.e. the units) with a hexagonal symmetry (Fig. 4a).13) The projected distance 113 Å is related with the 3D distance of 131 Å by a factor of cos30 ˚ (i.e. 0.866). Each column in Fig. 4b is a projection of not a single but a number of cylinders (e.g. A, B, C). Here it is important to note that the focus of any electron microscope is deep, when compared with the size of biomolecules, and thus an image obtained is not a section but a projection of the object.

**Fig. 5:**
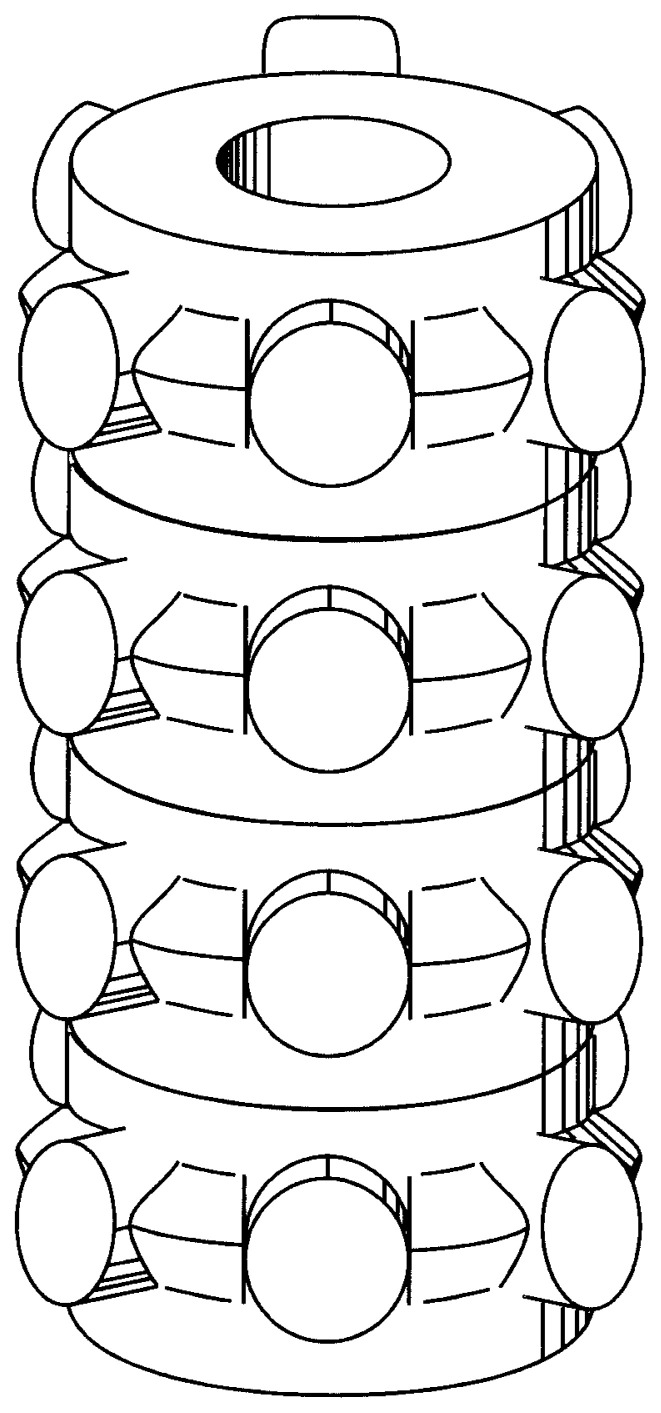
A schematic drawing of a possible 3D structure of the cylinder.

A 3D model of the cylinder predicted from the two views (Fig. 4a, b) is shown in Fig. 5, although for a precise reconstruction of a 3D structure EM images need to be combined in the Fourier space but not in the real space.3),4)

In general, FFRPs form various types of assemblies, and alternation between these forms is important for their gene regulation.11) The unit for forming any assemblies is a dimer, and the dimer structure is kept essentially the same.12) These facts provide us with a unique opportunity for combining EM with X-ray crystallography. Copies of an X-ray dimer structure can be assembled by following an EM-based scheme, such as Fig. 5. Various crystals of FFRPs, too small to be studied by X-ray crystallography, have been already obtained in addition to types of aggregates (Koike, H. *et al*., unpublished).

## Determination of the tilt angle

The periodicity 1/(113 Å) found in the Fourier transform of electron micrograph 1 (Fig. 2a) was found in another Fourier transform of electron micrograph 2, taken at the magnification 44.9 k (Fig. 2c), and also present in the electron diffraction (Fig. 2b). While, in Fig. 2a the vertical pair was identified at the spatial frequency of 1/(29 Å), but in Fig. 2c a similar pair was identified at 1/(13.5 Å), a frequency more than twice the other one. Various areas in the two micrographs were examined by calculating their Fourier transforms, but these were the only two frequencies, observed in the respective electron micrographs. No intermediate was found, unless both types were absent, as was the case for the electron diffraction (Fig. 2b), covering the largest area.

**Fig. 6:**
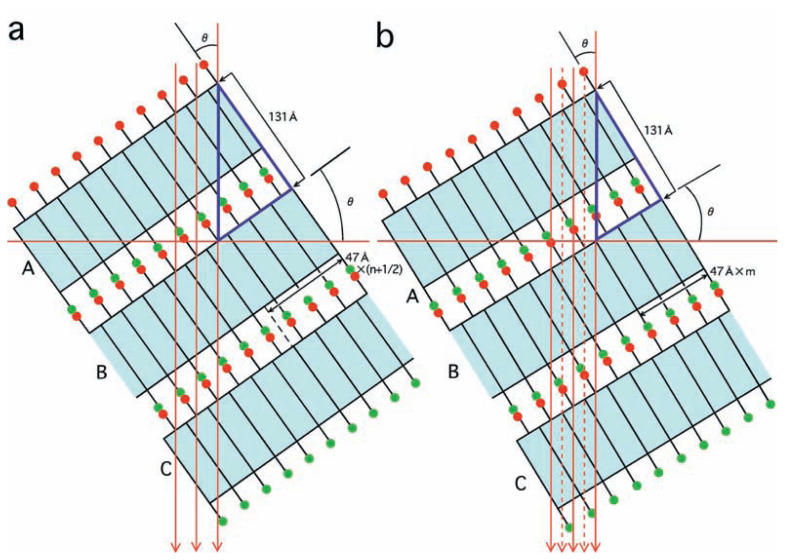
A schematic representation of tilting of cylinders A, B, C in the same layer (Fig. 4a) with different angles. Electrons proceed along the arrows. Note that on the two sides of e.g. cylinder B, one facing A and the other facing C, the phases are shifted by half the segment (indicated by relative positions of two types of linkers, red and green). In (a) units stacking inside each cylinder will be projected onto those in the other cylinders without shift (compare positions where the same arrow intersects with the three cylinders). In (b) units in cylinder B are projected by shifting, by half the periodicity, from those in A and C (compare positions where a broken arrow intersects with the three cylinders). The blue triangles show that *θ* needs to satisfy equations: tan*θ* = 47 × (n+1/2)/131 in (a) and tan*θ* = 47 × m/131 in (b).

One way to explain these observations is to assume that cylinders neighboring each other in the same layer (e.g. A and B, or B and C in Fig. 4a) are shifted from each other by half the disk unit along the fiber axis (Fig. 6), and that in the two electron micrographs cylinders are projected with slightly different tilts.

With an arbitrarily tilt, *θ*, the segmental structure of the projected cylinders will become obscure: the periodicity inside any cylinders is kept to h cos*θ*, where h is the length of segments in 3D, but the phases of cylinders A, B, C projected onto each other can be different. However, the segments will become clear, if those of each cylinder precisely overlay those in other cylinders with no relative shift (Fig. 6a). For this an equation needs to be satisfied: tan*θ*= (n + 1/2) × h/131, where n is an integer (see a blue triangle in Fig. 6a). Alternatively, another angle can be chosen, so that segments in cylinder B are shifted by half a unit length from those in A and C (Fig. 6b). The unit length in projection becomes half, (h/2)cos*θ*(see broken lines added in Fig. 6b), satisfying another equation, tan *θ*= m × h/131, where m is an integer.

From these sets of equations, for electron micrograph 1, *θ* is estimated to be 52 ˚, and n plus 1/2 is estimated as 3.5. While, for electron micrograph 2, *θ* is estimated as 55 ˚, and m is 3. In short, the two micrographs were taken with a very small difference in tilt. Alternatively, the tilt angle was changing slightly among the cylinders, and the ratios of the two subpopulations were slightly different in the two views. The real periodicity h is now calculated as 47 Å.

## The hidden helical symmetry

It is known from the X-ray structure12) that the cylinder is not an accumulation of discs but, in fact, it is helical: the term disks needs to be replaced by turns. This helicity was not directly identified in the EM images.

**Fig. 7:**
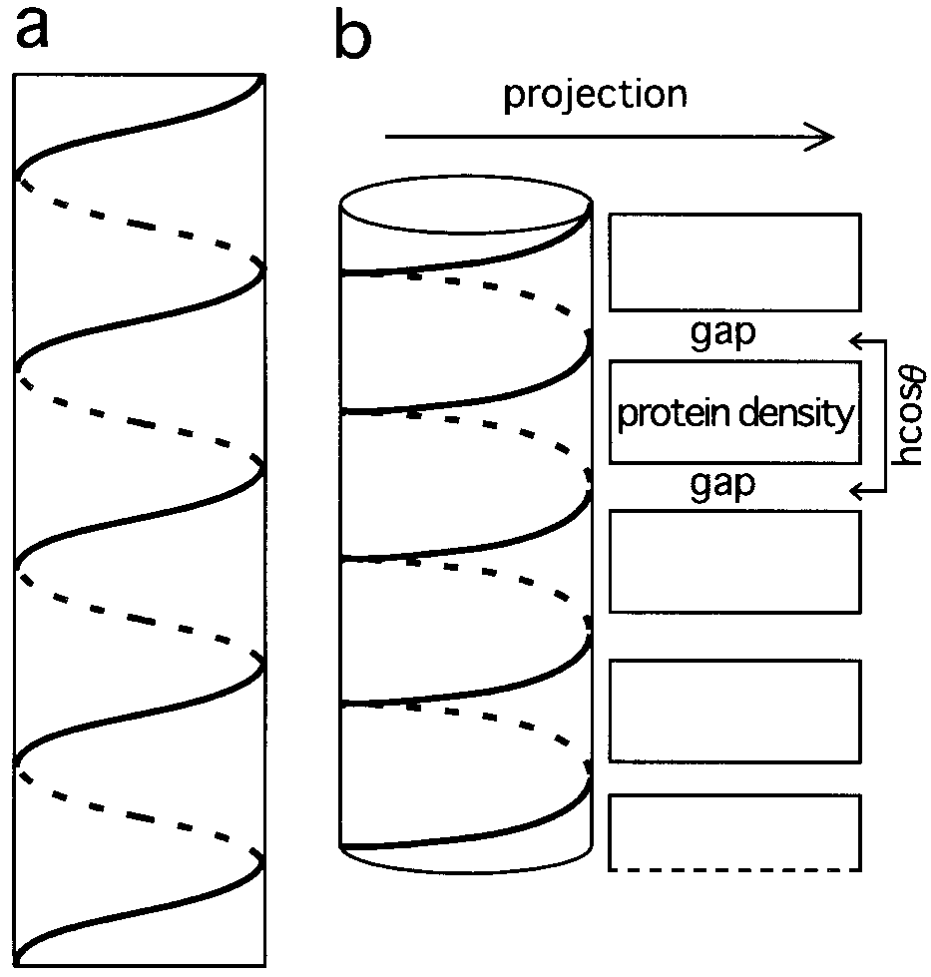
A schematic representation of helices projected with (b) or without (a) tilt. In (b) each projected helical turn (left) creates a density (indicated by a rectangle, right). Between such densities gaps are created. h: the helical pitch. *θ*: the tilt angle.

For a helical object, or its ideal projection, Fourier spots will be arranged into the shape of an X.25) The angle between the two bars crossing, when measured over the vertical axis, coincides with twice the tilt angle of the helix measured from the horizontal plane. When this angle is small, as is the case for the helix formed by DM1 dimers, in the Fourier space, “X” becomes similar to “I”. In addition, when the helix is tilted with an angle as large as 50 ˚, its projection is not completely sinusoidal (Fig. 7a), but becomes ladder-like (Fig. 7b left). Between higher densities of proteins projected to rectangles, empty spaces will be left (Fig. 7b right), creating a repetitive structure.

The only EM observation related with the helicity is the half phase shifting between cylinders, e.g. A and B, (Fig. 6) suggesting that each ring has different phases on its two sides. However, it would be difficult to conclude a helicity solely from this observation, unless one learns the answer from the X-ray structure. The only way to identify the hidden helicity by EM appears to be to obtain another side view with a smaller tilt.
